# Solution structure of the Drosha double-stranded RNA-binding domain

**DOI:** 10.1186/1758-907X-1-2

**Published:** 2010-01-12

**Authors:** Geoffrey A Mueller, Matthew T Miller, Eugene F DeRose, Mahua Ghosh, Robert E London, Traci M Tanaka Hall

**Affiliations:** 1 Laboratory of Structural Biology, National Institute of Environmental Health Sciences, National Institutes of Health, Research Triangle Park, NC, USA

## Abstract

**Background:**

Drosha is a nuclear RNase III enzyme that initiates processing of regulatory microRNA. Together with partner protein DiGeorge syndrome critical region 8 (DGCR8), it forms the Microprocessor complex, which cleaves precursor transcripts called primary microRNA to produce hairpin precursor microRNA. In addition to two RNase III catalytic domains, Drosha contains a C-terminal double-stranded RNA-binding domain (dsRBD). To gain insight into the function of this domain, we determined the nuclear magnetic resonance (NMR) solution structure.

**Results:**

We report here the solution structure of the dsRBD from Drosha (Drosha-dsRBD). The αβββα fold is similar to other dsRBD structures. A unique extended loop distinguishes this domain from other dsRBDs of known structure.

**Conclusions:**

Despite uncertainties about RNA-binding properties of the Drosha-dsRBD, its structure suggests it retains RNA-binding features. We propose that this domain may contribute to substrate recognition in the Drosha-DGCR8 Microprocessor complex.

## Background

MicroRNA (miRNA) are small regulatory RNAs derived from longer RNA transcripts called primary miRNA (pri-miRNA) ([1], reviewed recently in [2]). Pri-miRNA are cleaved by an RNase III family enzyme called Drosha to produce hairpin precursor miRNA (pre-miRNA) [3]. Pre-miRNA are transported to the cytoplasm [4-7] and further processed by Dicer enzymes to produce mature miRNA [8-13]. Drosha contains two RNase III domains that form the enzyme's catalytic center. At the C-terminus is a double-stranded RNA-binding domain (dsRBD), which is essential for pri-miRNA processing [14].

To process pri-miRNA, Drosha forms an enzyme complex with a partner protein DiGeorge syndrome critical region 8 (DGCR8; also known as Pasha in *Drosophila *and *Caenorhabditis elegans*) [14-17], which contains two dsRBDs. DGCR8 has been proposed to be a crucial factor for recognition of pri-miRNA substrate via its dsRBDs [18]. A crystal structure of the tandem dsRBDs of DGCR8 revealed closely interacting domains whose conformation would not be expected to change upon RNA binding [19]. A model for RNA recognition suggests that the two domains bind to portions of the pri-miRNA that are distant from each other. It is not known whether the dsRBD of Drosha is also important for substrate RNA binding or serves another function, since little to no RNA-binding activity has been observed for Drosha and the dsRBD is not necessary for interaction with DGCR8 [14,18,20,21]. To gain insight into the function of Drosha-dsRBD, we determined the solution structure of this domain. The structure suggests it retains RNA-binding features. We suggest this domain may participate in RNA interaction with DGCR8 in the context of the microprocessor complex.

## Results and Discussion

**Figure 1:**
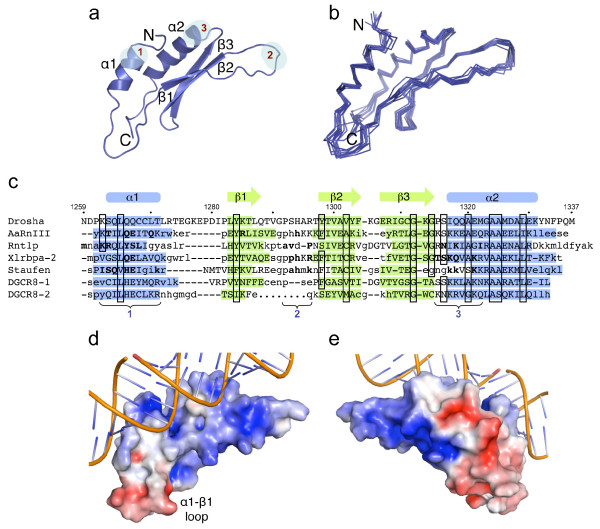
**Nuclear magnetic resonance (NMR) solution structure of Drosha-double-stranded RNA-binding domain (dsRBD)**. **(a) **Ribbon diagram of the lowest energy-minimized structure of Drosha-dsRBD. Regions of dsRBDs that typically interact with RNA are highlighted with light blue and labeled as in the text. **(b) **Superposition of the Cα traces of the 10 lowest energy-minimized structures of Drosha-dsRBD. **(c) **Structure-based sequence alignment of Drosha-dsRBD and selected dsRBDs. Amino acid residues that do not structurally align with Drosha-dsRBD are shown in lower case letters. Secondary structural elements and amino acid numbers for Drosha-dsRBD are indicated. Boxed residues are well conserved among dsRBDs. Typical RNA-interacting regions are indicated with brackets, and RNA-interacting residues are in bold. Sequences of *Aquifex aeolicus *RNase III (AaRnIII) [24], *Saccharomyces cerevisiae *Rnt1p [23,35], *Xenopus laevis *Xlrbpa-2 [26], *Drosophila melanogaster *Staufen dsRBD-3 [25], and DiGeorge syndrome critical region 8 (DGCR8) protein dsRBD 1 (DGCR8-1) and 2 (DGCR8-2)[19] are shown. **(d, e) **Electrostatic surface representation calculated using the APBS package [36] of the lowest energy-minimized structure of Drosha-dsRBD. Red (negative) is set at - 3 kT/e and blue (positive) is set at 3 kT/e. RNA is from *A. aeolicus *RNase III in complex with dsRNA substrate [PDB:2EZ6]. Panel e is rotated 180° relative to the other panels. This figure was prepared with the PyMol package [37].

**Figure 2:**
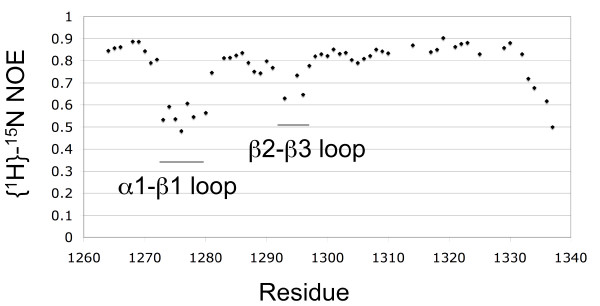
**Heteronuclear nuclear Overhauser effect (NOE) {^1^H}-^15^N measured at 14.1 T**. The ratio of measured intensity with and without presaturation is plotted versus residue, for those residues with well isolated (non-degenerate) chemical shifts.

The solution structure of Drosha-dsRBD comprises an α helix (Ser1263 to Thr1271), followed by three β strands forming an antiparallel β sheet (Leu1283 to Gly1314), and terminating with a second α helix (Ile1317 to Lys1331) (Figure 1a-c). This αβββα fold is consistent with the core structures of other members of the dsRBD family [22]. Residues highly conserved among dsRBDs and important for the fold are found in the Drosha-dsRBD (boxed in Figure 1c) [22]. A unique feature of the Drosha-dsRBD is an extended α1-β1 loop. This loop is compact in all other known dsRBD structures. The α1-β1 loop shows some of the lowest {^1^H}-^15^N-nuclear Overhauser effects (NOEs) (Figure 2), indicating it is dynamic on a fast time scale (picoseconds to nanoseconds).

Sequence features important for RNA recognition are also conserved in Drosha-dsRBD. In structures of dsRBDs in complex with RNA, the domain binds to one face of a dsRNA helix, and three regions are important for RNA recognition: β1 (region 1), the β1-β2 loop (region 2), and the β3-α2 loop (region 3) [22]. Helix α1 and the β1-β2 loop interact with successive minor grooves of the dsRNA, and the β3-α2 loop interacts with the intervening major groove. RNA interacting residues in region 1 are conserved in Drosha-dsRBD. For example, Lys1262 is equivalent to Lys271 in *Saccharomyces cerevisiae *Rnt1p, which contacts the RNA substrate, and mutation of Rnt1p-Lys271 to alanine severely suppresses *in vivo *RNA processing [23]. This lysine residue is conserved in dsRBDs associated with RNase III enzymes. Similarly, Gln1267 is equivalent to *Aquifex aeolicus *RNase III Glu158, Rnt1p Ser376, *Xenopus laevis *Xlrbpa-2 Glu119, and *Drosophila melanogaster *Staufen Glu7, which contact the RNA backbone [23-26]. In region 2, His1294 and Arg1296 are equivalent to His141 and Arg143 in Xlrbpa-2, which contact the RNA in the subsequent minor groove [26]. A cluster of basic and polar side chains in region 3 typically contacts the major groove. Drosha-dsRBD lacks a high density of basic residues in this region (Figure 1c); thus it is possible that interactions with the major groove are minimal or comprise mainly polar interactions.

The distribution of charged side chains on the surface of the protein is also consistent with RNA binding (Figure 1d, e). A positively charged region could facilitate the binding of the negatively charged phosphate backbone of an RNA molecule. This region extends to the opposite side of the dsRBD, which is not the typical RNA-binding surface. We superimposed Drosha-dsRBD and the dsRBD of *A. aeolicus *RNase III [24] to illustrate how Drosha-dsRBD could bind to a dsRNA (Figure 1d, e). Given the electrostatic surface and the presence of specific RNA-interacting residues, Drosha-dsRBD appears capable of binding RNA, despite the inability to demonstrate interaction of Drosha or its dsRBD with pri-mRNA [18,20,21]. From the model in Figure 1d, the extended α1-β1 loop in Drosha-dsRBD could interact with the RNA, adding a new substrate recognition feature. However, the loop is negatively charged, and although this does not exclude nucleic acid interaction [27,28], alternatively it could facilitate intermolecular or intramolecular protein-protein interaction. Both this loop and the β1-β2 loop are not positioned to allow direct interactions with the straight, regular RNA duplex in the model. The substrates of Drosha are hairpin pri-miRNA with mismatched and bulged bases that would form irregular structures. Thus the substrate RNA could be bent and the protein loops could alter conformation to allow interaction.

DGCR8 contains two dsRBDs, which recognize pri-miRNA [18-20]. In the crystal structure of the tandem dsRBDs of DGCR8, the dsRBDs likely bind to separate dsRNA regions on the pri-miRNA [19]. Pri-miRNA contain long hairpin loops with several distinguishing characteristics: The 5' and 3' ends are unstructured basal segments, an approximate 11-bp lower stem proceeds from the basal segments to the cleavage site, and on the other side of the cleavage site is an approximate 22-bp upper stem that ends with a terminal loop [18]. These features are important for substrate recognition and/or cleavage site location [21,29]. The reported affinity of DGCR8 for pri-miRNA is relatively weak (*K*_d _= 2 mM) [19], and full-length Drosha or Drosha-dsRBD exhibit poor, if any, binding to RNA on their own [18,20,21]. Perhaps the Drosha-DGCR8 complex has greater affinity and specificity with each dsRBD fine tuning substrate recognition by binding to a specific feature of the pri-miRNA. For example, the dsRBDs of DGCR8 may recognize the upper and lower stem regions of the pri-miRNA near the basal segments and terminal loop, respectively, while Drosha-dsRBD may bind to the central region near the cleavage site, as is observed with *A. aeolicus *RNase III [24]. Additional biochemical and structural studies are needed to understand fully how each dsRBD participates in substrate recognition. Such studies would benefit from abundant pure Drosha/DGCR8 complex.

## Conclusions

We have determined the solution structure of Drosha-dsRBD. The structure is similar to other RNA-binding dsRBDs, and features important for RNA recognition are conserved. A long loop between α1 and β1 is unique to Drosha-dsRBD. We propose Drosha-dsRBD may participate in RNA recognition in the Drosha-DGCR8 complex, despite little to no RNA binding on its own.

## Methods

### Protein expression and purification

Human Drosha-dsRBD [EMBL:AF189011] (amino acids 1,259 to 1,337, Addgene plasmid no. 108,208; Addgene, Cambridge, MA, USA) was expressed with a C-terminal His_6 _tag using pET21c(+). Protein was expressed in BL21(DE3) cells induced with 1 mM isopropyl β-D-1-thiogalactopyranoside (IPTG) for 3 to 5 h at 37°C. Single-labeled and double-labeled proteins were generated by growth in M9 minimal media including combinations of ^13^C-labeled glucose and/or^15^N-labeled ammonium chloride. Protein was purified using Ni^2+^-NTA resin followed by separation on a Resource Q anion exchange column (GE Healthcare, Uppsala, Sweden). For nuclear magnetic resonance (NMR) analysis, the purified protein was pooled and exchanged into a buffer comprising 25 mM tris(hydroxymethyl)aminomethane (Tris)(D_11_) pH 7.0, 100 mM KCl, 1 mM dithiothreitol (DTT)(D_10_), 1 mM ethylenediaminetetraacetic acid (EDTA), 10% D_2_O, and 10 mm dimethylsilapentanesulfonate (DSS) by concentration and dilution.

### NMR structure determination and refinement

Standard triple resonance NMR experiments were utilized to assign the backbone and side-chain resonances of the proteins [30]. Proton chemical shift assignment was 86% complete as assessed by the CYANA package [31]. Of note, the only NOE experiment acquired was the 4D simultaneous ^13^C/^15^N nuclear Overhauser enhancement spectroscopy (NOESY) [32]. Customized scripts were written to unalias peaks and write formatted files for structure calculation and automated assignment with CYANA. The structures were subsequently refined with the XPLOR-NIH package [33] utilizing the hydrogen bond distance angle (HBDA) module [34] for hydrogen bond restraints. Structural statistics are given in Table 1.

**Table 1:** Structural statistics for the 10 lowest energy-minimized conformers of Drosha-double-stranded RNA-binding domain (dsRBD)

Statistic	Value
NOE distance restraints:	
Intraresidue	98
Sequential	111
Medium range (i, i + 2 to 4)	74
Long range (i, i>4)	206
Total	489
Dihedral restraints:	189
Hydrogen bond (HBDA):	27
Ensemble RMSD:	
Backbone secondary structure	0.44
Heavy atoms secondary structure	0.86
Violations:	
NOE	0
Dihedral	0
HBDA	1.4
RMS experimental:	
NOE	0.02 ± 0.005
Dihedral	0.415 ± 0.077
HBDA	0.006 ± 0.006
RMS covalent geometry:	
Bonds	0.003 ± 0.000
Angles	0.514 ± 0.009
Impropers	0.362 ± 0.013
Ramachandran space:	
Most favored region	81.6%
Additionally allowed	17.0%
Generously allowed	1.4%
Disallowed	0

HBDA = hydrogen bond distance angle; NOE = nuclear Overhauser effect; RMSD = root mean square deviation.

### Accession numbers

The PDB coordinates [PDB:2KHX] and NMR assignments (16,256) have been deposited to the Research Collaboratory for Structural Bioinformatics (RCSB) and BioMagResBank (BMRB) databases, respectively.

## Competing interests

The authors declare that they have no competing interests.

## Authors' contributions

GAM designed, oversaw and performed data acquisition and analysis, interpreted the data, and participated in drafting the manuscript. MTM conceived the study, expressed and purified the protein, performed data acquisition and analysis, and participated in drafting the manuscript. EFD performed data acquisition and analysis. MG assisted with sample preparation and performed data acquisition and analysis. REL designed and oversaw data acquisition and analysis. TMTH oversaw the study, interpreted and analyzed the data, and drafted the manuscript. All authors read and approved the final manuscript.
